# Who is respectful? Effects of social context and individual empathic ability on ambiguity resolution during utterance comprehension

**DOI:** 10.3389/fpsyg.2015.01588

**Published:** 2015-10-23

**Authors:** Xiaoming Jiang, Xiaolin Zhou

**Affiliations:** ^1^Center for Brain and Cognitive Sciences and Department of Psychology, Peking UniversityBeijing, China; ^2^School of Communication Sciences and Disorders, McGill UniversityMontréal, QC, Canada; ^3^Key Laboratory of Machine Perception and Key Laboratory of Computational Linguistics (Ministry of Education), Peking UniversityBeijing, China; ^4^Beijing Key Laboratory of Behavior and Mental Health, Peking UniversityBeijing, China; ^5^IDG McGovern Institute for Brain Research at PKU, Peking UniversityBeijing, China

**Keywords:** social status, pragmatics, referential ambiguity, directly-quoted utterance, pronoun resolution, ERP

## Abstract

Verbal communication is often ambiguous. By employing the event-related potential (ERP) technique, this study investigated how a comprehender resolves referential ambiguity by using information concerning the social status of communicators. Participants read a conversational scenario which included a minimal conversational context describing a speaker and two other persons of the same or different social status and a directly quoted utterance. A singular, second-person pronoun in the respectful form (*nin*/*nin-de* in Chinese) in the utterance could be ambiguous with respect to which of the two persons was the addressee (the “*Ambiguous* condition”). Alternatively, the pronoun was not ambiguous either because one of the two persons was of higher social status and hence should be the addressee according to social convention (the “*Status* condition”) or because a word referring to the status of a person was additionally inserted before the pronoun to help indicate the referent of the pronoun (the “*Referent* condition”). Results showed that the perceived ambiguity decreased over the *Ambiguous, Status*, and *Referent* conditions. Electrophysiologically, the pronoun elicited an increased N400 in the *Referent* than in the *Status* and the *Ambiguous* conditions, reflecting an increased integration demand due to the necessity of linking the pronoun to both its antecedent and the status word. Relative to the *Referent* condition, a late, sustained positivity was elicited for the *Status* condition starting from 600 ms, while a more delayed, anterior negativity was elicited for the *Ambiguous* condition. Moreover, the N400 effect was modulated by individuals' sensitivity to the social status information, while the late positivity effect was modulated by individuals' empathic ability. These findings highlight the neurocognitive flexibility of contextual bias in referential processing during utterance comprehension.

## Introduction

Establishing referential relations is vital to verbal communication (Brown-Schmidt and Hanna, 2011). Verbal expressions are often ambiguous, particularly in supportive contexts. Considering a situation when *John met his friend Bob and Shawn. John asked “which course are you going to teach next semester?”* As a third-party observer, one may be confused as to whom John is addressing without the addressee being explicitly referred to in the speech. However, if one knows that Bob is a lecturer and Shawn is a student at the university, one may immediately infer that Bob is the target addressee. The addressee or the observer may employ a variety of information from the context to resolve this temporary referential ambiguity, building up representation for the utterance as it unfolds over time.

Among context information, the social status of the speaker and the addressee has been demonstrated to be a significant cue relevant to attention, perception, decision-making, and inference-making (Dalmaso et al., 2012, 2014; Hu et al., 2014; Mason et al., 2014; Koski et al., 2015) and is linguistically marked in certain languages (e.g., the second-person pronoun in Mandarin, French, Spanish etc.). The social status of communicators is typically realized by cues related to job titles and professions (e.g., *professor*) which are attained by individuals involved in the conversation and which form a set of features that are uniquely associated with high vs. low status (Koski et al., 2015). The linguistic marker such as, *nin*/*nin-de* (you/your), a respectful form of the second-person pronoun in Mandarin Chinese, is normally used by a lower-status speaker to address a higher-status addressee; in contrast, *ni/ni-de* (you/your), an informal version of the second-person pronoun, is typically used by a lower-status speaker to address a lower-status and/or familiar addressee. Our previous work (Jiang et al., 2013b) demonstrated that a mismatch of the social status between the addressee and the respectful/informal form of the pronoun elicits neural responses associated with the perception of deviance, including N400, P600, and late negativity (N600) effects in event-related potentials (ERPs). A successful resolution of referential ambiguity associated with social/pragmatic information may require accessing information from long-term memory, holding multiple pieces of information in working memory, and making use of complex inference procedures (Brown-Schmidt and Hanna, 2011). A critical question is how the brain uses social status information concerning the communication partners in resolving referential ambiguities and how these processes may vary between individuals with differential social abilities during utterance comprehension.

### Social context and referential ambiguity

Behavioral and neurophysiological studies have implicated that listeners use both discourse and social contexts to resolve referential ambiguity during language comprehension. The discourse context biases the interpretation of the addressee and affects the neural responses underlying ambiguity resolution on the noun (Nieuwland et al., 2007) and pronoun (Nieuwland and Van Berkum, 2006). A frontal sustained negativity effect (Nref) was observed on the ambiguous pronoun, the gender of which was congruent with two competing antecedents in the context, relative to the pronoun referring specifically to one antecedent. This effect was reduced when contextual information (e.g., verb) biased one antecedent to be more probable than the other (e.g., “*The chemist hit the historian when he…”*), or was completely absent when a discourse context implied the death or leaving of one antecedent from the discourse (Nieuwland et al., 2007). These findings suggest that the context-based pragmatic inference reduces both ambiguity in referential processing and the neural activity underlying this processing. Such ambiguity-related neural responses are also modulated by the comprehenders' working memory span, with higher span comprehenders exhibiting stronger responses (Nieuwland and Van Berkum, 2006).

Evidence from eye-tracking studies has also revealed that the shared knowledge and beliefs between the speaker and addressee provide constraints on the resolution of referential ambiguity (Keysar et al., 1998; Hanna et al., 2003; Barr, 2008; Brown-Schmidt and Tanenhaus, 2008; Heller et al., 2008; Brown-Schmidt, 2009; Ferguson and Breheny, 2012; Bezuidenhout, 2013; Ferguson et al., 2015), resulting in different eye gaze patterns on the object displayed in a shared perspective vs. the object in an addressee-privileged perspective, when the object was referred to in speech. In tasks involving a real conversation, the communication partners coordinated on an object-matching task for a display of objects. The target object referred to in the speaker's instruction was accompanied by an object competing in their initial phonological structure (e.g., *bucket/buckle*, Barr, 2008) or by an object with the same shape and color (e.g., two *blue triangles*, Hanna et al., 2003). The display of the target object was shared between the speaker and the addressee (the participant) and the display of the competitor was either shared or was only visible to the addressee (who possessed the knowledge that the speaker could not see this object; Barr, 2008). The frequency of fixation was equally deployed when the target and the competitor were in the shared perspective but was prioritized for the target object when the competitor was only in the addressee-privileged perspective (Hanna et al., 2003; cf. Barr, 2008), indicating that access to the other's perspective reduced the competitor interference effect in face of referential ambiguity.

Recent work on shared beliefs (e.g., Ferguson et al., 2015) required participants to watch a movie in which a character (*Jane*) either held a false or a true belief of an object's location while at the same time listening to a description (*Jane will look for the chocolates in the container on the left/right*) in which the character's belief resulted in ambiguity of the location (*container*) which could not be resolved until the end of the sentence. When asked to make an inference of the character's belief, the comprehenders' eye-movements were immediately guided to the object based on the false belief of the actor from the onset of the sentence (*Jane*); the comprehenders' eyes, however, were not fixated on the object until the sentence-final disambiguating word in an irrelevant task. These finding suggests that the successful inference of other's knowledge or perspective facilitates the resolution of referential ambiguity and this inference process is most likely cognitively effortful.

### Neurocognitive evidence of contextual effects and individual differences in pragmatic language processing

Other evidence has also demonstrated the contextual effect on the integration of an upcoming input word. Two ERP effects, an N400 and a late positivity, are mostly reported to vary as a function of contextual variables. A factual statement inconsistent with one's real-world knowledge (Hagoort et al., 2004) or with one's inference from a counter-factual construction (Nieuwland and Martin, 2012) elicited larger N400 responses. This N400 effect appears when a statement mismatched the cultural convention of the comprehender (“*Every single Welsh child can sing in* *tune*,” presented to a Welsh-speaking comprehender) but is absent when it is irrelevant (e.g., the same utterance presented to an English-speaking comprehender; Ellis et al., 2015). Morally-laden statements disagreeing with one's belief system elicit stronger N400 responses than agreeing statements (Van Berkum et al., 2009). The discourse context implying the positive or negative characteristic of a person affects the integration of this person's name in the subsequent sentence in which the name was positively or negatively valenced; the name incongruent with the context elicited a larger N400 or delayed positivity as compared with the congruent one, depending on the valence endowed with the name (Wang et al., 2015). An enlarged N400 was also present on words describing a character's emotional reaction which mismatched the expected feeling in a socio-emotional vignette (Leuthold et al., 2012). The N400 effect in these studies suggests an increased integration demand for unifying a word into a broad context, ranging from linguistic to social and extending to the comprehender's own knowledge or belief system.

The context is also a useful source of information for deriving non-literal interpretation. An utterance (“*Tonight we gave a superb performance”*) with a context facilitating an ironic interpretation (*Both ladies sang off key*) elicited an increased late positivity (P600), compared with an utterance containing only the literal interpretation (Spotorno et al., 2013). A similar positivity effect was observed on utterances presented with ironic vs. neutral-intending prosody (Regel et al., 2011), demonstrating a non-literal interpretation beyond linguistic input via pragmatic inference. The late positivity was preceded by an N400 when the non-literal expression was unfamiliar to the listener (Filik et al., 2014) or was constrained by minimal context (Coulson and Kutas, 2001). Some studies also reported a more sustained positivity, which was found on words inconsistent with the preceding context describing one's traits, intention, or goal of an action, indicating the comprehender's attempt to infer these implied messages (Van Duynslaeger et al., 2007; Baetens et al., 2011). This sustained effect is related to the activity of the neural network subserving the mentalizing process, including the temporoparietal junction (Van Duynslaeger et al., 2007). Jiang et al. (2013b) observed a sustaining positivity following N400 on respectful second-person pronouns (i.e., *nin-de*, your) incorrectly referring to a lower-status addressee as compared with the pronoun correctly referring to a higher-status addressee. This sustained positivity effect was interpreted as reflecting a second-pass reanalysis process, which resulted in a sarcastic interpretation of the input sentence. However, a sustaining negativity following N400 was elicited on a less respectful second-person pronoun (i.e., *ni-de*, your) incorrectly referring to a higher-status addressee as compared with the pronoun correctly referring to a lower-status addressee. This negativity effect was interpreted as reflecting a second-pass inhibitory process when no sarcasm could be derived from the input (as such derivation would violate social norms).

Individuals' characteristics, such as empathic ability, modulate language use, and the neural activity underlying the pragmatic processes. Differential neural responses have been revealed between individuals with autism spectrum disorders (ASD) and healthy individuals during pragmatic language comprehension (Tesink et al., 2009): individuals with ASD showed stronger activations in the right inferior frontal gyrus when comprehending speech violating the voice-inferred speaker's social status and an absence of activation in the ventromedial prefrontal cortex in comprehending speaker-consistent speech. Moreover, eye-tracking studies using the visual-world paradigm suggested that the perspective of a communication partner is immediately taken into account by the listener when interpreting what was said, especially in determining what was referred to in the context (e.g., Ferguson et al., 2010; Brown-Schmidt and Hanna, 2011). These findings highlight the role of perspective taking in utterance comprehension. A third-party's interpretation of directly-quoted utterances between communication partners may involve perspective-taking that allows the comprehender to take the speaker's or the addressee's perspective. Recent ERP evidence suggests that empathy and its sub-processes modulate the use of contextual information and its effect on the integration of upcoming information. Scalar sentences such as *some people have lungs* in which the critical word “lungs” did not match the pragmatic interpretation of the scalar quantifiers (i.e., only some of the people have lungs) elicited a larger N400 as compared with the counterpart word in felicitous sentences (e.g., “pets” in *some people have* *pets*; Nieuwland et al., 2010).

Such neural responses are also modulated by individuals' autistic quotient (AQ, Baron-Cohen et al., 2001), an index inversely correlated with one's empathic ability. Nieuwland et al. (2010) split the group of participants based on the median AQ score and observed an N400 effect only for individuals having lower AQ (i.e., higher empathic ability). Using an empathy questionnaire (Baron-Cohen and Wheelwright, 2004), Van den Brink et al. (2012) demonstrated that the increased N400 responses in the mismatch of speaker identity and speech content (*I cannot sleep with my* *teddy bear* *in my arm*, spoken in an adult male voice) was only observed in listeners with higher empathic ability; participants with lower empathic ability, in contrast, showed a positivity effect.

Individuals' empathic ability also modulates ERP responses to status-mismatch on the second-person pronoun (Jiang and Zhou, 2015). The N400 effect was only observed in participants displaying higher fantasizing ability as measured by the Interactive Reactivity Index (Davis, 1983). Moreover, the cognitive components of empathic ability, as measured by IRI, modulated the neural activity underlying the interpretation of sentences with pragmatic under specification or pragmatic failure (Li et al., 2014). The fantasizing ability (to imagine oneself to be the character of a novel or movie) affected the activation in the medial prefrontal cortex when a description of an event was underspecified (and hence requiring pragmatic inference), suggesting the deployment of mentalizing process to infer a proper representation of the event satisfying the pragmatic constraints. The perspective-taking ability (to shift one's perspective to that of the other) affected the activation in the bilateral inferior frontal gyrus when the description of an event mismatched the comprehender's knowledge about the likelihood of the event. These findings suggest that cognitive empathy could be linked to the individual's ability in using contextual information and making pragmatic inference during verbal communication.

### The present study

We aim to investigate when and how a comprehender, as a third-party, resolves referential ambiguity in a conversation scenario by using information concerning the social status of communicators in the context, and how his/her empathic ability and sensitivity to the social status information modulates ambiguity perception and the underlying neural activity. The comprehender's empathic ability was measured using the empathy score (40-items) in Baron-Cohen and Wheelwright (2004); the status sensitivity was defined as the difference in rating the appropriateness of status-incongruent and status-congruent scenarios on a 7-point scale (on a subset of stimuli from Jiang et al., 2013b). Participants were asked to explicitly rate the ambiguity of scenarios depicting social interaction involving interpersonal communication and to read these scenarios for comprehension while undergoing EEG recording. We created scenarios in Mandarin Chinese which included a context introducing a speaker of lower social status and two potential addressees with the same (the ambiguous context, in the *Ambiguous* condition) or different social status (the status-biased context, in the *Referent* and *Status* conditions). In both the *ambiguous* and *Status* conditions, a directly-quoted utterance began with the respectful form of the Chinese second-person pronoun (*nin*/*nin-de*). This pronoun was referentially ambiguous in the *ambiguous* condition because both the potential addressees were of equally high social status and hence both could be the target, but was not ambiguous in the *Status* condition because the social convention concerning the use of the respectful form would predict the person of higher status to be the target. The status of the potential addressees was indicated clearly in the context by a status word used together with the family name (e.g., *Professor Wu*). Finally, the *Referent* condition differed from the *Status* condition in that a status word indicating a higher-status/position (such as *Professor, General, Boss*, etc.), consistent with one of the status words used in the context, was inserted before the pronoun to additionally indicate which one of the two persons in the context should be the target addressee.

Behaviorally, we predicted a reduction of ambiguity rating for the Referent and Status conditions as compared with the Ambiguous condition, due to a successful matching of the referent and the pronoun in the Status situation and the additional information conveyed by the status word in the Referent condition. On the ERPs time-locked to the pronoun, we would normally predict an Nref effect for the ambiguous condition. The Nref is a sustained negativity that starts at about 300 ms and lasts for several hundreds of milliseconds (Van Berkum et al., 1999). This effect, distributing mainly at anterior sites, appears when two antecedents are equally suitable, rendering the interpretation of the pronoun ambiguous. It has been claimed to reflect the detection of ambiguity, the controlled process of ambiguity resolution, and/or the maintenance of two referential interpretations in working memory (Van Berkum et al., 2007). However, the present study did not have the unambiguous, baseline condition in which there was only one antecedent in the context. Although the pronoun in the *Status* or the *Referent* condition was unambiguous, the interpretation of this pronoun in these conditions came with a processing cost that may have overshadowed the potential Nref effect for the *Ambiguous* condition, especially in the early time window (see below).

We predicted increased N400 responses for the *Referent* condition, as compared with the *Status* condition. Although adding a status word before the second-person pronoun in the *Referent* condition would mark even more clearly who is the referent of the pronoun, the pronoun has to nevertheless be integrated with both the status word and the targeted addressee. This integration is perhaps more resource-demanding than the integration just between the pronoun and the referent. Moreover, we would also predict an N400 effect for the *Status* condition, as compared with an unambiguous, single-referent baseline condition if the latter were included in the design. Using pragmatic information to infer (and select) the referent of a pronoun from the two potential candidates and linking the pronoun with the referent would be more difficult than simply linking a pronoun with a single candidate in the context, resulting in increased N400 responses (c.f., Jiang et al., 2013b). This prediction would lead us to compare the *Status* with the *ambiguous* condition, which might yield no or small differences in the early time window as both the potential N400 and Nref effects were in the same direction.

For the late time windows, we predicted that the Nref effect for the *Ambiguous* condition, as compared with the *Referent* condition, would eventually be detectable. This was because the Nref effect in processing the ambiguous pronoun in the *Ambiguous* condition would last for a long time, whereas the processing cost for integrating the pronoun with the status word and the targeted addressee in the *Referent* condition would have already dissipated by this time. In contrast, we predicted an increased late positivity for the *Status* condition, relative to the *Referent* condition. To link the pronoun with one of the potential referents in the context, a pragmatic inference process must take place to decide which person was of higher status and hence could be addressed with the respectful form. Previous studies have shown that this inference process is usually accompanied by the late positivity (e.g., Jiang et al., 2013b).

As we indicated above, in the *Status* condition, to decide which of the two addressees should be the referent of the pronoun, a pragmatic inference process must occur. This process may vary as a function of the comprehender's empathic ability. The higher the ability, the more successful the inference process. Moreover, comprehenders with increased sensitivity to the social status information should find the sentences less ambiguous when the status information is relevant for a successful inference for the referent of the pronoun (in the *Status* condition) and should find the sentences even more ambiguous when no such information is available (in the *Ambiguous* condition).

Given the previous findings of the modulation of empathy on language comprehension (Nieuwland et al., 2010; Van den Brink et al., 2012; Li et al., 2014; Jiang and Zhou, 2015), we predicted that the magnitude of the N400 effect in the *Referent* condition may be modulated by individuals' empathic abilities. The status sensitivity may also affect the N400 responses because integration of the current pronoun into the preceding context depends on the matching of the status information between the antecedent and the pronoun in all the conditions. Moreover, both empathic ability and status sensitivity could modulate the late positivity effect given that the pragmatic inference process in selecting a likely addressee would depend highly on one's ability to use this information in the context.

## Method

### Participants

Thirty-two right-handed university students (22 females, aging from 18 to 28 years, mean age = 21.2 years) gave informed consent to participate in the ERP experiment. All the participants were native Mandarin speakers born and raised in Beijing. They spoke the Beijing dialect of Mandarin and had not lived outside of the Beijing area before college. This selection criteria was used to ensure that the participants were sensitive to the use of the respectful form of the second person pronoun, since some Mandarin dialects do not use this form. All the participants had normal or corrected-to-normal vision and none had reported reading impairment or any type of neurological or psychiatric disorders. This study was carried out in accordance with the Declaration of Helsinki and was approved by the Ethics Committee of the Department of Psychology, Peking University.

### Design and material

One hundred and sixty triplets of scenarios describing events in daily life were created, from which 150 triplets were selected as the critical material (Table 1). Each scenario comprised a directly-quoted utterance and a conversational context preceding the utterance. The conversational context described a daily situation in which one character was meeting or interacting with the other two characters. For all the scenarios, the first character always served as the speaker and one of the other two characters as the addressee. The social status was conveyed by the name of each character, which consisted of a common Chinese family name which had no status meaning (e.g., *Li, Zhang, Yang*, etc.) and a position name which conveyed a particular level of social status in the social hierarchy (e.g., higher-status: *Professor, General, Manager*, etc.; lower-status: *Student, Soldier, Assistant*, etc.). The status level of each name was pre-evaluated by a university student who speaks the Beijing dialect. The speaker in a scenario was always in lower status. The addressees were of different status in the Referent and Status conditions, with one higher than the other; the addressees were of equal status in the Ambiguous condition, with both addressees holding higher status than the speaker. For the scenario with addressees of different status, the higher-status addressee preceded (i.e., was mentioned earlier than) the lower-status one in half of the scenarios and was preceded by the lower-status addressee in the other half.

**Table 1:** **Examples of conversational scenarios used in the experiment**.

Referent	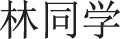	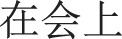	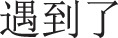	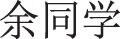	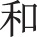	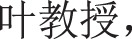	
	Student Lin	on the conference	met	Student Yu	and	Professor Ye,	
	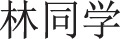	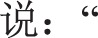	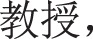	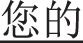	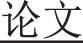	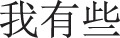	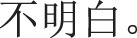
	Student Lin	said,”	Professor,	your (nin-de)	article	I have some	questions.”
	Student Lin met student Yu and professor Ye on the conference. Student Lin said, “Professor, I have some questions about your _[respectful]_ article.”						
Status	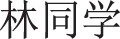	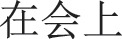	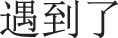	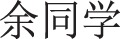	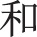	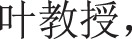	
	Student Lin	on the conference	met	Student Yu	and	Professor Ye,	
	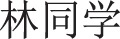	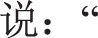	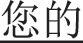	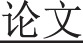	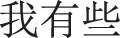	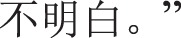	
	Student Lin	said,”	your (nin-de)	article	I have some	questions.”	
	Student Lin met student Yu and professor Ye on the conference. Student Lin said, “I have some questions about your _[respectful]_ article.”						
Ambiguous	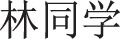	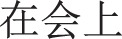	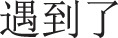	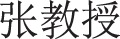	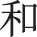	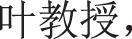	
	Student Lin	on the conference	met	Professor Zhang	and	Professor Ye,	
	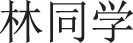	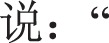	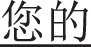	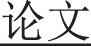	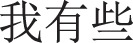	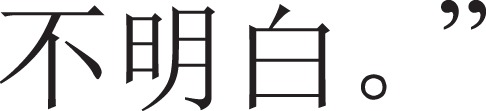	
	Student Lin	said,”	your (nin-de)	article	I have some	questions.”	
	Student Lin met student Yu and professor Ye on the conference. Student Lin said, “I have some questions about your _[respectful]_ article.”						

*Critical pronouns and the object nouns are underlined*.

Each utterance was composed of an object-subject-verb (OSV) structure beginning with either a status/position noun (e.g., Professor) which stood for the addressee in the *Referent* condition, or a singular, respectful-form of the second-person possessive pronoun (i.e., *nin-de*) in the *Status* and *Ambiguous* conditions. The utterance delineated an action that the speaker performed for the addressee, a message to the addressee, or the speaker's attitude toward the addressee. The same possessed object (e.g., *article* in the exemplars in Table 1) was used across the three conditions. All the objects were status-neutral, which were equally likely to be possessed/ owned by a higher- or lower-status person. The predicates used in the utterance were also status-neutral.

One hundred and twenty unambiguous scenarios were created as fillers to prevent the use of potential response strategies, with 80 scenarios involving 3 characters (1 speaker and 2 addressees) and 40 scenarios involving 2 characters (1 speaker and 1 addressee). Among these fillers, 40 scenarios were created with the same context sentences as those in the *Ambiguous* condition, but began the utterance with a status/position name which unambiguously stood for the addressee; this was to eliminate the potential strategy of anticipating an unspecified pronoun when reading the ambiguous context. To eliminate the strategy of anticipating a higher-status person to be the addressee in comprehending status-biased context, another 40 scenarios were composed of contexts with a higher-status speaker and two characters of different status levels, but the utterances began with either the plain form of the second-person pronoun (*ni-de/your*) referring to the low-status addressee or a status word referring to one of the addressees (20 scenarios for each). Thus, the addressee was not predictable until the status word or the second-person pronouns was revealed. The remaining 40 two-character scenarios were selected from Jiang et al. (2013b) which included characters in different social status and a pronoun in its singular, respectful (*nin-de*, your) or in a singular, informal form (*ni-de*, your), referring to the addressee at a certain status level in the scenario.

### Scenario rating

The scenarios were selected from a larger sample of 160 sets of scenarios based on a reference ambiguity rating prior to the ERP experiment which aimed to examine the ability of the utterance-initial pronoun to refer unambiguously to a person in the multi-character conversational context. The 160 sets of scenarios were created using the same criteria as those described for the critical scenarios, and were divided into three lists using a Latin-square procedure. Thirty native speakers of Beijing Mandarin (21 females, aging from 18 to 26 years, mean age = 21.8 years) who were not tested for EEG took part in this pretest (Table 2). They were randomly assigned to one of the three lists (each with 10 participants) and were instructed to rate the level of ambiguity of the pronoun in referring to an antecedent in the context (1 = the most ambiguous, and 7 = the least ambiguous). To minimize the potential referential bias due to the information following the pronominal phrase, an incomplete utterance was given (e.g., in the *Referent* condition, *Student Lin met student Yu and Professor Ye in the conference. Student Lin said, “Professor, your…*”).

**Table 2:** **Mean ambiguity rating scores in two independent groups of participants in the pretest and the post-EEG test**.

**Experimental condition**	**Pretest**	**Post-EEG test**
*Referent*	6.81 (0.13)	6.90 (0.24)
*Status*	5.21 (1.04)	5.36 (1.06)
*Ambiguous*	1.73 (0.66)	1.81 (1.09)

The ambiguity rating was based on a seven-point Likert scale, with 7 representing “the least ambiguous” and 1 representing “the most ambiguous.”

The critical sets of scenarios were selected to ensure that the rating for the chosen scenarios was the lowest for the *Referent* condition and the highest for the *Ambiguous* condition. ANOVA with Scenario Type as a within-participant factor revealed a main effect of scenario type, *F*1_(2, 58)_ = 412.28, *p* < 0.001, ɳ${}_{p}^{2}=0.93$; *F*2_(2, 298)_ = 1572.20, *p* < 0.001, ɳ${}_{p}^{2}=0.91$, with the lowest level of ambiguity for the *Referent* condition (Mean = 6.81, *SD* = 0.13), followed by the *Status* condition (Mean = 5.21, *SD* = 1.04), and the highest for the *Ambiguous* condition (Mean = 1.73, *SD* = 0.66). The differences between conditions were all significant, *p*s < 0.001 (see Table 2).

### Procedure

Participants were seated comfortably in a sound-proofed and electronically shielded chamber. They were instructed to move their head or body as little as possible and to keep their eyes fixated on a sign at the center of the computer screen before the onset of each scenario. The fixation sign was at eye-level and was approximately 1 m away. Scenarios were presented segment-by-segment in a rapid serial visual presentation (RSVP) mode at the center of the screen, with less than 1 degree of horizontal visual angle and 0.2 degree of vertical angle for one segment to minimize the eye-movement. Each scenario consisted of a series of eight frames (Table 1). Each segment was presented in a comfortable rate of 400 ms followed by a blank screen of 400 ms. Participants were asked to read scenarios carefully for comprehension. At the end of each scenario, participants were presented with a probe statement and were asked to verify whether the statement was consistent with the information described in the scenario. The statement could probe constituents in the context, including the speaker and the location of the conversation/interaction (e.g., for *Technician Wang met Technician Zhang and Director Li in the office, Wang said, “Director, I have achieved the goal,”* the probe was *Technician Wang met Direct Li at the metro station*), or constituents in the directly-quoted utterance, including the actor, the patient, and the verb (e.g., for *Student Dong encountered Student Chen and Madam Chu, Dong said, “Madam, your story touches me so much,”* the probe was *Madam Chu was touched*). This task did not facilitate the reader to access the social status information in the conversational context but required a certain level of comprehension of the directly-quoted utterance (Regel et al., 2010; Jiang et al., 2013b). Each condition required the same numbers of consistent (“yes”) and inconsistent (“no”) responses. Participants were asked to respond as accurately as possible by pressing a button on a joystick with their right index fingers. Each probe statement was presented 1200 ms after the offset of the last segment of the scenario and remained on the screen until the participants made a decision. The next trial began 1000 ms after button press. Participants were randomly assigned to one of the three experimental lists, created using a Latin Square procedure. For each list, scenarios were pseudo-randomized so that no more than three consecutive scenarios were from the same critical condition, no more than three consecutive scenarios were followed by a statement probing the same constituents in the scenario, and no more than three scenarios were followed by the same “yes” or “no” response. A practice session of 14 scenarios were presented to each participant prior to the experiment.

A few behavioral measurements were administered after the EEG session. Participants were asked to complete the Empathy Quotient (EQ-40) questionnaire to measure self-reported empathic abilities (Baron-Cohen and Wheelwright, 2004). A Chinese version of the reading span task adapted from Daneman and Carpenter (1980) was used to measure verbal working memory performance (Ye and Zhou, 2008). Two scenario rating tests were administered to all the participants to validate the contextual manipulation and to evaluate individual differences in the sensitivity to the social status information in the context. In the reference ambiguity rating, participants were asked to rate (7-point Likert scale, 1-representing the most ambiguous and 7-representing the least ambiguous) the level of ambiguity of a given pronoun referring to a person in the conversational context (i.e., the same as the pretest) for all the critical stimuli. In the appropriateness rating, participants rated the degree of appropriateness of using a pronoun (7-point Likert scale, 1-representing the least appropriate and 7-representing the most appropriate). Included were four types of scenarios, including 10 containing the correct use of and 10 containing the incorrect use of the respectful form of the second-person pronoun (*Nin-de*, a higher-status speaker addressing a lower-status addressee) and 10 containing the correct use of and 10 containing the incorrect use of the informal form of the second-person pronoun (*Ni-de*, a lower-status speaker addressing a higher-status addressee).

### EEG recording

EEGs were recorded from 64 scalp sites using Ag/AgCl electrodes mounted in an elastic cap (Brain Products, Munich, Germany) according to the international 10–20 system. The vertical electro-oculogram (VEOG) was recorded supra-orbitally from the right eye. The horizontal EOG (HEOG) was recorded from electrodes placed at the outer canthus of the left eye. All EEGs and EOGs were referenced online to an external electrode placed on the tip of nose and were re-referenced offline to the mean of the bilateral mastoids. Electrode impedance was kept below 5 kΩ for all electrodes. The bio-signals were amplified with a band pass from 0.016 to 100 Hz and digitized online with a sampling frequency of 500 Hz.

### EEG analysis

The EEG data were preprocessed with Brain Vision Analyzer software. The EEG signals were corrected for ocular artifacts using algorithms developed by Gratton et al. (1983), and were then segmented with an epoch of 1800 ms time-locked to the onset of the pronoun (from 200 ms before to 1600 ms after the onset). The segmented EEGs were filtered with a 30 Hz low-pass filter with a slope of 24 dB/oct. The resulting data were baseline corrected according to the mean amplitude of the activity pre-onset of the stimuli (−200 to 0 ms). Trials were rejected if they exceeded ± 70 μV in amplitude, contained a transient of over 100 μV in a period of 100 ms, or contained activity lower than 0.5 μV in a period of 100 ms.

Trials that were inaccurately verified and contaminated by excessive artifacts were excluded from the statistical analysis, rendering 33, 37, and 38 trials on average for the *Referent, Status*, and *Ambiguous* conditions, respectively. The differences between conditions were not significant, *F* < 1. Mean ERP amplitudes were calculated for each time window, participant, and condition. Based on visual inspection and previous findings on the respectful pronoun (Jiang et al., 2013b), four time windows of interest were selected: 300–600 ms for the N400, 600–900 ms for the late positivity, 900–1600 ms for the sustained late positivity, and 1300–1600 ms for the sustained anterior negativity. Repeated-measures ANOVA was performed on the mean amplitudes, with experimental conditions (3 levels: *Referent, Status, Ambiguous*), Hemisphere (3 levels: left, medial, right), and Region (3 levels: anterior, central, posterior) as within-participant variables. The Hemisphere and Region were crossed, forming nine regions of interest (ROI), each with 5–6 representative electrodes: left-anterior (F7, F5, F3, FT7, FC5, FC3), left-central (T7, C5, C3, TP7, CP5, CP3), left-posterior (P7, P5, P3, PO7, PO3), medial-anterior (F1, Fz, F2, FC1, FCz, FC2), medial-central (C1, Cz, C2, CP1, CPz, CP2), medial-posterior (P1, Pz, P2, O1, POz, O2), right-anterior (F4, F6, F8, FC4, FC6, FT8), right-central (C4, C6, T8, CP4, CP6, TP8), and right-posterior (P4, P6, P8, PO4, PO8). Mean ERP magnitudes for each ROI were averaged over the electrodes in each region.

To evaluate the effects of empathic ability and status-sensitivity on pronoun resolution in each condition, these ANOVA models also included EQ or Differential Score between status-incongruent and status-congruent sentences in the post-EEG Appropriateness Rating (as an index of status-sensitivity) as a covariate. WM span was added as a statistical control. Regression analysis was further performed on each ERP effect whenever there was an interaction involving experimental condition and EQ/Differential Score, using EQ or Differential Score as an independent factor and the magnitude difference in an ERP effect as a dependent factor. All the continuous variables were z-score transformed before entering the model. Greenhouse-Geisser correction was applied whenever the degree of freedom was above 1. *Post-hoc* comparisons between conditions were planned and the significance level was estimated with Bonferroni correction. Partial ɳ^2^ was reported as a measure of effect size (ɳ${}_{p}^{2}$). Marginally significant effects were further examined with Bayesian Factor (BF), which was calculated as the ratio between the probability of an effect to be true and the probability of a null effect based on the observation (Morey and Rouder, 2011; Rouder et al., 2012), and were only considered more likely to be true when the BF was larger than three (Rouder et al., 2009). The reported marginal effects all survived this examination.

## Results

### Individual differences measures

The post-ERP questionnaire revealed large individual differences in both EQ (Mean = 39.63 out of 80, ranging from 16 to 61) and WM span (Mean = 3.19 out of 7, ranging from 2 to 6.5). No correlation was observed between EQ and WM span, *r* = 0.01, *p* = 0.94.

### Post-ERP scenario ratings

For the appropriateness rating, the repeated-measures ANOVA included Scenario Type (*Status-congruent* vs. *Status-incongruent*) as a within-participant factor for by-participant and by-item analysis and EQ as a covariate for by-participant analysis. To control for the effect of WM on pronoun resolution (Nieuwland and Van Berkum, 2006, 2008), we included WM span as a control variable in all the by-participant analyses. The ANOVA revealed a significant effect of Scenario Type, *F*1_(1, 29)_ = 219.18, *p* < 0.001, ɳ${}_{p}^{2}=0.88$, *F*2_(1, 19)_ = 447.85, *p* < 0.001, ɳ${}_{p}^{2}=0.96$. Consistent with Jiang et al. (2013b), the appropriateness rating showed that participants rated the status-incongruent utterances (3.14 for *Nin-de* sentences and 2.49 for *Ni-de* sentences) as less appropriate than status-congruent utterances (6.49 for *Nin-de* sentences and 6.65 for *Ni-de* sentences), suggesting that the participants were sensitive to the social status information in the context and were aware of the misapplication of pronoun to an addressee of a certain social status. The by-participant analysis revealed a significant interaction between EQ and congruency, *F*_(1, 29)_ = 3.11, *p* = 0.03, ɳ${}_{p}^{2}=0.77$. A linear regression analysis revealed that empathy only modulated the appropriateness rating in the congruent condition, *b* = 0.15, *t* = 2.10, *p* = 0.04, indicating that participants with higher empathy tended to judge the congruent sentences to be more appropriate than those with lower empathy (6.67 vs. 6.44 out of 7, if participants were median split and grouped according to the scores of the empathy measure).

Consistent with the rating prior to the EEG experiment, the post-EEG ambiguity rating showed that the participants rated the *Referent* condition as the least ambiguous (Mean = 6.89, *SD* = 0.25), the *Status* condition as more ambiguous (Mean = 5.37, *SD* = 1.06), and the *Ambiguous* condition as the most ambiguous (Mean = 1.79, *SD* = 1.09). ANOVAs were conducted, taking experimental condition (3 levels: *Referent, Status, Ambiguous*) as a within-participant factor for by-participant and by-item analysis and EQ as a covariate for by-participant analysis. Results revealed a significant main effect of experimental condition, *F*1_(2, 58)_ = 237.35, *p* < 0.001, ɳ${}_{p}^{2}=0.89$; *F*2_(2, 298)_ = 4146.59, *p* < 0.001, ɳ${}_{p}^{2}=0.97$. *Post-hoc* comparisons showed that the differences between conditions were all significant, *p*s < 0.0001. The by-participant ANOVA also revealed a significant interaction between condition and EQ, *F*_(2, 58)_ = 3.56, *p* = 0.02, ɳ${}_{p}^{2}=0.09$. The regression analysis revealed a marginally significant effect of EQ on the rating score in the *Status* condition, *b* = 0.36, *t* = 2.05, *p* = 0.05, suggesting that participants with higher empathy tended to judge the sentences to be less ambiguous. The scores were 5.42 vs. 5.09 (out of 7) for the median split groups.

To further analyze the effect of individual sensitivity to status information on the ambiguity rating, we performed ANOVA including experimental condition as a within-participant factor and the Differential Score in the post-EEG appropriateness rating (calculated for each participant) as a covariate. Results revealed a significant interaction between Scenario Type and Differential Score, *F*_(2, 58)_ = 25.38, *p* < 0.001, ɳ${}_{p}^{2}=0.47$. The ambiguity rating was positively predicted by Differential Score in the *Status* condition, *b* = 0.55, *t* = 3.53, *p* < 0.005, and in the *Referent* condition, *b* = 0.08, *t* = 1.95, *p* = 0.06, and negatively predicted by Differential Score in the *Ambiguous* condition, *b* = −0.73, *t* = −4.60, *p* < 0.005. These findings suggested that the larger the difference the participant showed in the appropriateness ratings (i.e., the more status-sensitive), the less ambiguous they judged the scenarios in the Referent and Status conditions, and the more ambiguous they judged the scenarios in the Ambiguous condition. The rating scores were 6.94 vs. 6.83, 5.69 vs. 4.91, and 1.37 vs. 2.28 (out of 7) for the three conditions, respectively, if the participants were median split into the more sensitive vs. less sensitive group.

### Online sentence verification task

On average, 82.5% (*SD* = 9.9%), 84.4% (7.6%), and 85.0% (8.5%) scenarios were verified accurately for the *Referent, Status*, and *Ambiguous* conditions, respectively. No differences were found in accuracy between conditions, *F* < 1, suggesting that the participants were equally attentive to each type of scenario in the experiment.

### ERPs

**Figure 1:**
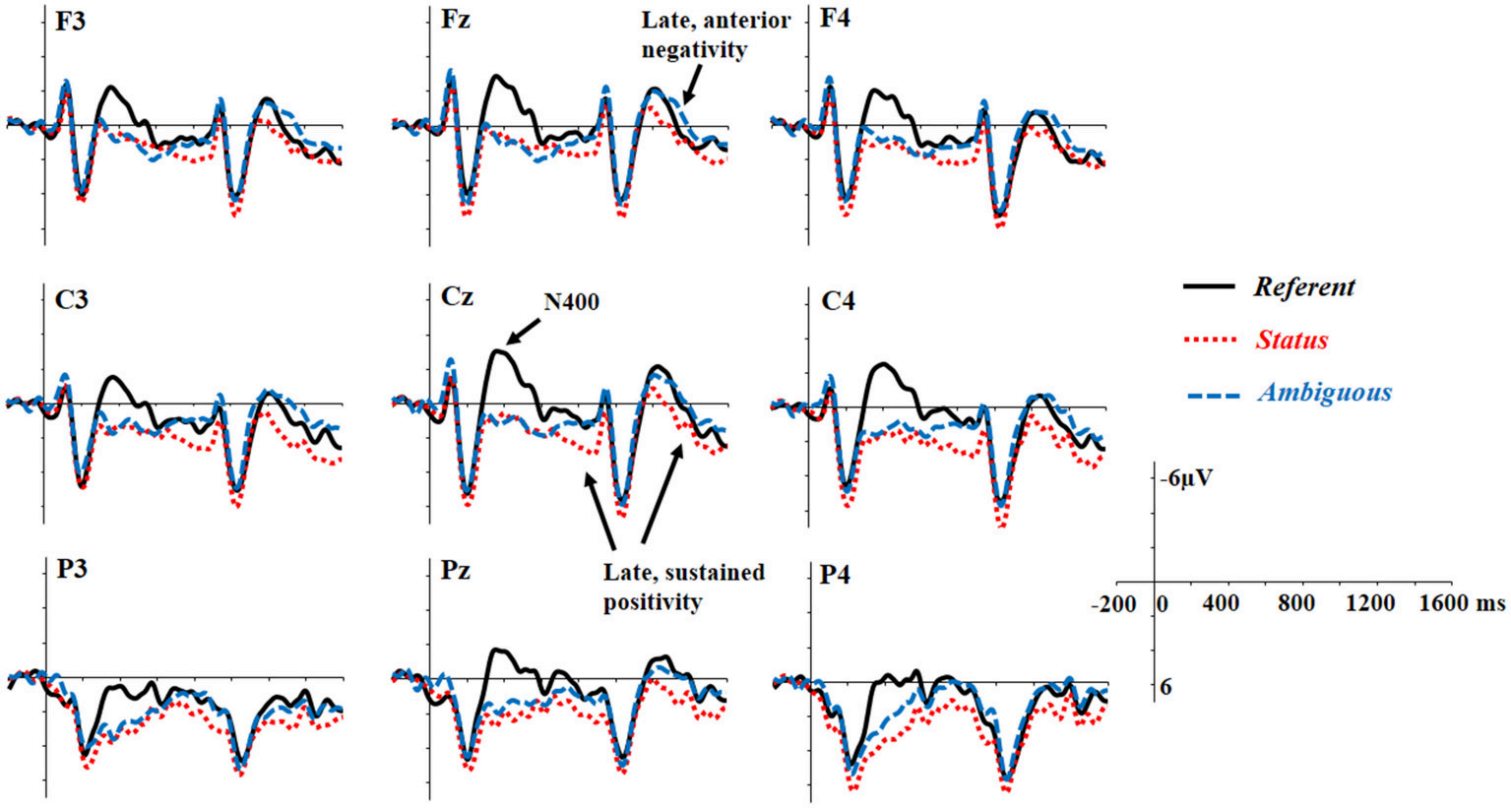
**Grand average waveforms time locked to the pronoun on 9 representative electrodes for the three critical conditions**.

**Figure 2:**
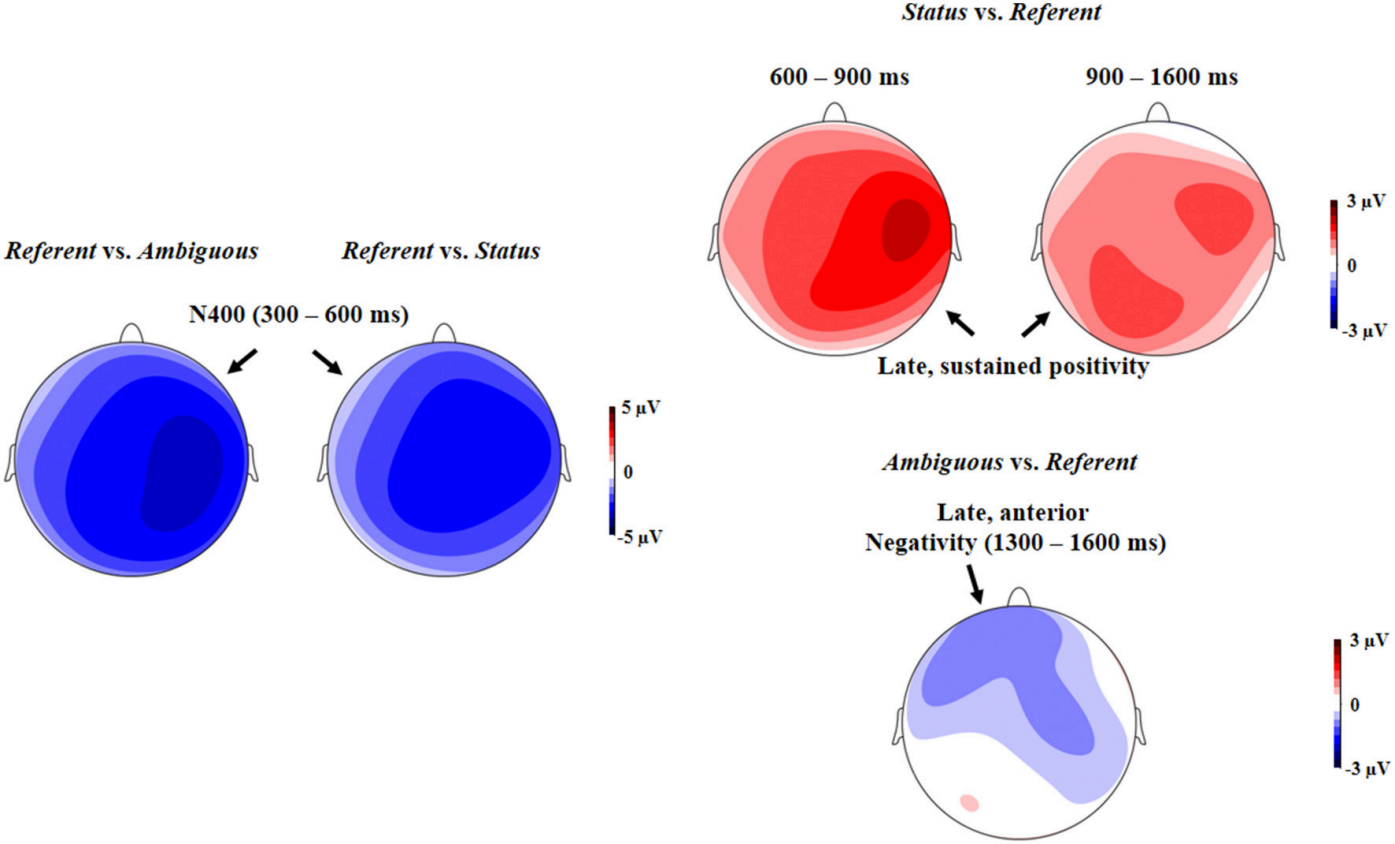
**Topographic maps showing the ERP differences from 300 to 600 ms (N400) between the *Referent* and the *Status* and between the *Referent* and the *Ambiguous* conditions (the left panel), the ERP differences between the *Status* and the *Referent* conditions in the time windows from 600 to 900 ms and from 900 to 1600 ms (the middle panels), and the ERP differences between the *Ambiguous* and the *Referent* conditions from 1300 to 1600 ms (the right panel)**.

Figure 1 depicts the grand average ERPs spanning from the pronoun to the following noun. The topographic distributions of the differential ERPs between conditions are displayed in Figure 2. The *Referent* condition elicited more negative responses (the N400 effect) in the 300–600 ms time window as compared with the *Status* and the *Ambiguous* conditions. Starting from around 600 ms, however, the *Status* condition showed more positive responses than the referent condition, and this positivity effect lasted until the end of the following noun (i.e., 1600 ms post onset of the pronoun). In contrast, in a later 900–1600 ms window, the *Ambiguous* condition showed an anteriorly distributed sustained negativity effect relative to the *Referent* condition. Statistical analyses confirmed these observations.

#### The N400 effect in the 300–600 ms time window

ANOVA with experimental condition (3 levels: *Referent, Status, Ambiguous*) and topographic variables (3 levels of Hemisphere: left, medial, right; 3 levels of Region: Anterior, Central, Posterior) as within-participant factors and EQ as a covariate revealed a significant main effect of condition, *F*_(2, 58)_ = 8.66, *p* = 0.001, ɳ${}_{p}^{2}=0.23$, with the *Referent* condition eliciting more increased N400 responses than the *Status* or the *Ambiguous* conditions, *p* < 0.001 and *p* < 0.05, respectively. No difference was found between the latter two conditions, *p* > 0.1. A significant interaction between experimental condition and Hemisphere was found, *F*_(4, 116)_ = 4.73, *p* < 0.01, ɳ${}_{p}^{2}=0.14$. As can be seen in Figure 2, the N400 effect for the *Referent* condition was larger in the right hemisphere than in the left hemisphere. There was also a four-way interaction between Scenario Type, Hemisphere, Region, and EQ, *F*_(8, 232)_ = 4.03, *p* < 0.01, ɳ${}_{p}^{2}=0.12$.

**Figure 3:**
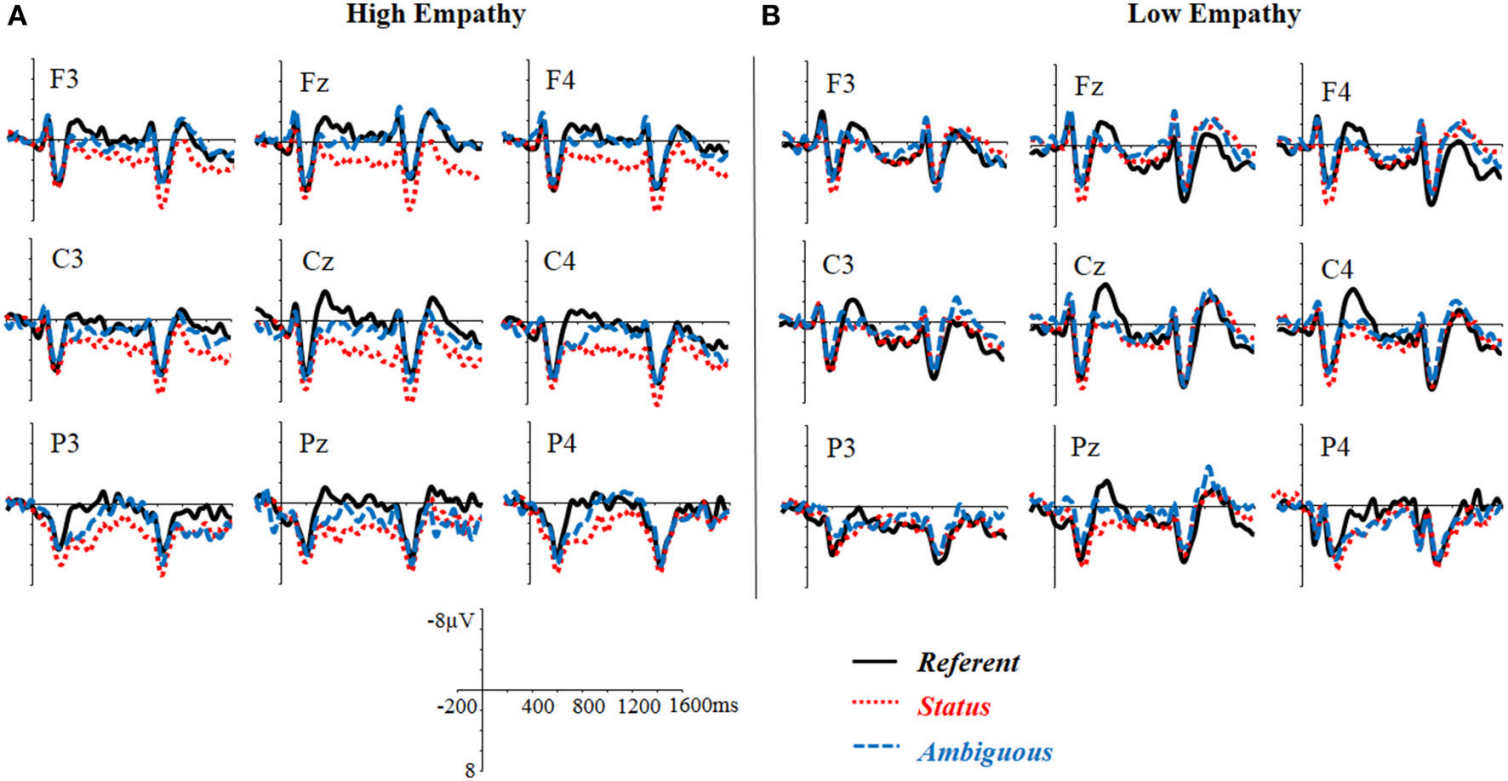
**Grand average waveforms time locked to the pronoun on 9 representative electrodes for the three critical conditions in the high-empathy (A) and the low-empathy (B) individuals**. The high- and the low-empathy individuals were defined according to the median split of the empathy score (Median = 39). Those with EQ lower than 39 were defined as low-empathy individuals (*n* = 14, Mean = 30.43, ranging from 16 to 37) while those with EQ higher than 39 were defined as high-empathy individuals (*n* = 15, Mean = 48.33, ranging from 41 to 61). Three individuals with EQ equal to 39 were not included in the figure.

To evaluate the relationship between EQ and the N400 effect, linear regression analyses were performed on each ROI, treating EQ as a covariate and WM as a control variable. The EQ significantly predicted the N400 difference between the *Referent* and the *Status* conditions in the left posterior region, *b* = −1.33, *t* = −2.91, *p* = 0.007, and the N400 difference between the *Referent* and the *Ambiguous* conditions in the left posterior region, *b* = −1.02, *t* = −2.41, *p* = 0.02. Participants with higher empathy tended to show larger N400 effects for the *Referent* condition relative to the *Status* and *Ambiguous* conditions (or more reduced N400 responses for the *Status* and *Ambiguous* conditions relative to the *Referent* condition). To illustrate this trend, we grouped participants according to their EQ scores and depict the group ERP responses in Figures 3A,B. It should be noted that, the N400 response in the *Status* condition, although much more reduced in the high-empathy group, may not represent what is typically meant by an N400-effect (Figure 3A). The high-empathy group showed a positive shift for the *Status* condition starting around 300 ms in the frontal region.

**Figure 4:**
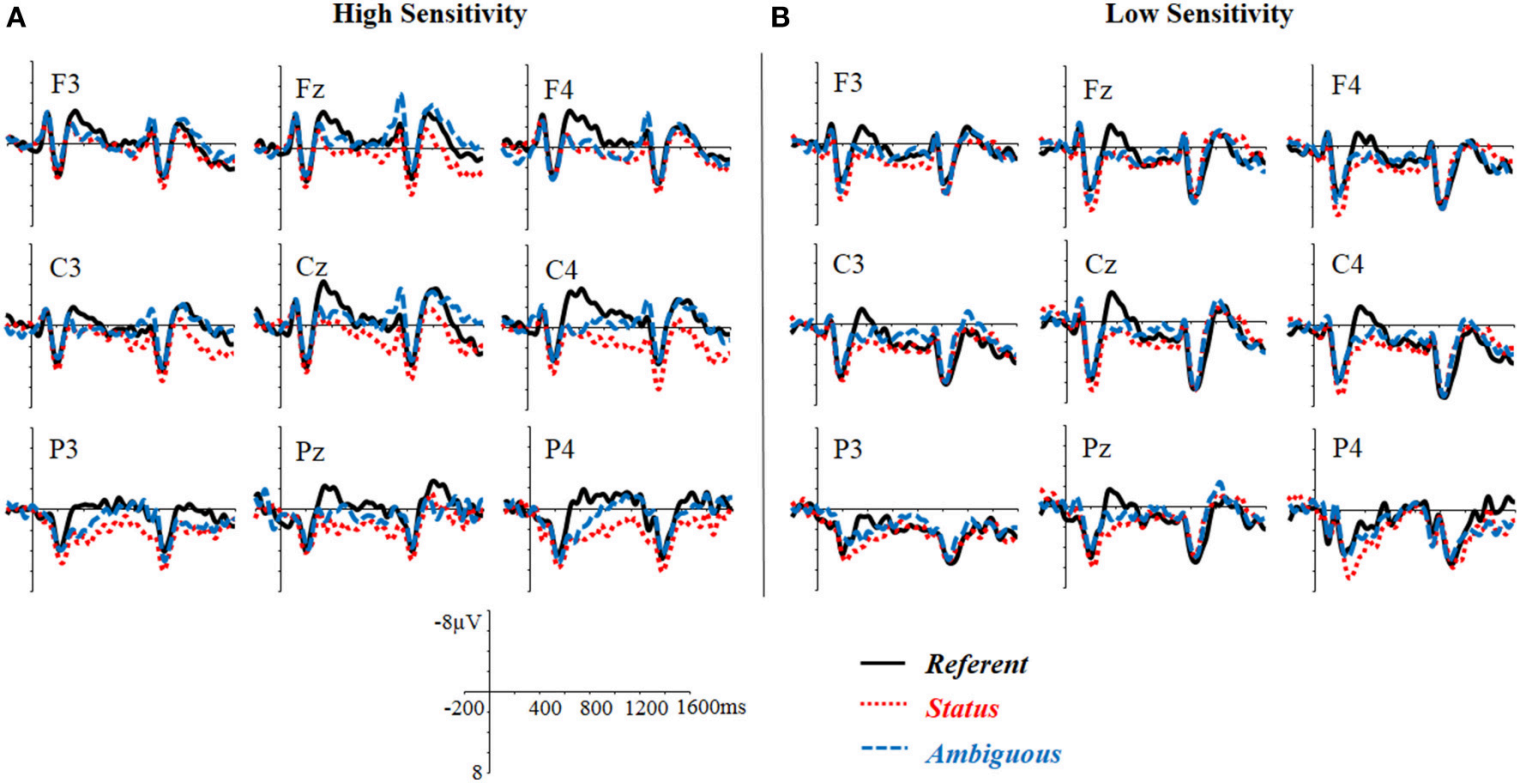
**Grand average waveforms time locked to the pronoun on 9 representative electrodes for the three critical conditions in the high-status-sensitivity (A) and the low-status-sensitivity (B) individuals**. The high- and the low-sensitivity individuals were defined according to the median split of the differential score (DS) in the appropriateness rating between the status-congruent and the status-incongruent condition (Median = 3.98). Those with the DS lower than 3.98 were defined as low-sensitivity individuals (*n* = 16, Mean = 2.71, ranging from 0 to 3.90) while those with DS higher than 3.98 were defined as high-sensitivity individuals (*n* = 16, Mean = 4.72, ranging from 4.05 to 5.95).

ANOVA with experimental condition (3 levels: *Referent, Status, Ambiguous*) and topographic variables (3 levels of Hemisphere: left, medial, right; 3 levels of Region: Anterior, Central, Posterior) as within-participant factors and the Differential Score in the appropriateness rating as a covariate revealed a significant three-way interaction between Differential Score, Scenario Type, and Hemisphere, *F*_(4, 116)_ = 3.11, *p* < 0.05, ɳ${}_{p}^{2}=0.10$. Regression analysis in each hemisphere, which controlled for WM, revealed a significant effect of Differential Score in the right hemisphere for the N400 differences between the *Referent* and the *Status* conditions and between the *Referent* and the *Ambiguous* conditions, *b* = −0.75, *t* = −2.55, *p* = 0.01; *b* = −0.78, *t* = −2.90, *p* = 0.005, respectively. These findings suggest that, distinct from the role of empathy, which predicted the N400 effect in the left and medial posterior regions, the status-sensitivity predicted this negativity effect in the right hemisphere. Participants who displayed increased sensitivity to the difference between the status-incongruent and the status-congruent scenarios had a larger N400 effect for the *Referent* condition (or more reduced N400 responses for the *Status* and the *Ambiguous* conditions, Figures 4A,B). Similar to the high-empathy group, the high-sensitivity group also showed a less typical pattern of N400 responses in the *Status* condition, with a positive shift following the negative peak at about 300 ms (Figure 4A).

#### The late positivity effects in the 600–900 ms window

ANOVA with experimental condition (3 levels: Referent, Status, Ambiguous) and topographic variables (3 levels of Hemisphere: left, medial, right, and 3 levels of Region: Anterior, Central, Posterior) as within-participant factors and EQ as a covariate revealed a significant main effect of condition, *F*_(2, 58)_ = 3.71, *p* < 0.05, ɳ${}_{p}^{2}=0.12$, indicating that the *Status* condition elicited a positivity effect relative to the *Referent* and *Ambiguous* conditions (Figure 2), *p*s < 0.05. No difference was observed between the *Referent* and the *Ambiguous* conditions, *p* > 0.1. There was a significant three-way interaction between experimental condition, EQ, and region, *F*_(4, 116)_ = 3.23, *p* < 0.05, ɳ${}_{p}^{2}=0.10$. Linear regression revealed a significant influence of EQ on the magnitude of the difference between the *Status* and the *Referent* conditions in all the regions (anterior: *b* = 1.03, *t* = 3.24, *p* = 0.002; central: *b* = 0.90, *t* = 2.58, *p* = 0.01; posterior: *b* = 1.00, *t* = 3.17, *p* = 0.002). These findings suggest that empathy predicted the late positive effect in the *Status* condition. The higher the empathic ability the participant exhibited, the larger the late positive effect (Figures 3A,B).

ANOVA with experimental condition (3 levels: *Referent, Status, Ambiguous*) and topographic variables (3 levels of Hemisphere: left, medial, right; 3 levels of Region: Anterior, Central, Posterior) as within-participant factors and Differential Score as covariate revealed a significant two-way interaction between Differential Score and experimental condition, *F*_(2, 58)_ = 3.09, *p* < 0.05, ɳ${}_{p}^{2}=0.09$. Regression analysis, which was performed on ERP differences collapsing over hemispheres and regions, revealed a significant effect of EQ on the ERP difference between the *Status* and the *Referent* conditions, *b* = 0.94, *t* = 4.93, *p* < 0.001. These findings suggest that the Differential Score in the appropriateness rating predicted the late positivity effect in the 600–900 ms time window: participants showing an increased sensitivity to the appropriate usage of pronoun in a status-given context also had larger late positivity for the *Status* condition (Figures 4A,B).

#### The delayed, sustained positivity effect in the 900–1600 ms time window

ANOVA taking experimental condition (3 levels: *Referent, Status, Ambiguous*) and topographic variables (3 levels of Hemisphere: left, medial, right; 3 levels of Region: Anterior, Central, Posterior) as within-participant factors and EQ as a covariate revealed a significant three-way interaction between Scenario Type, Hemisphere, and Region, *F*_(8, 232)_ = 2.68, *p* < 0.05, ɳ${}_{p}^{2}=0.09$. Further analysis on each ROI revealed a significant difference between the *Status* and the *Ambiguous* conditions in the left posterior, medial posterior, right central, and right posterior regions, *ps* < 0.05, and a significant difference between the *Status* and the *Referent* condition in the right central and right posterior regions, *ps* < 0.05, suggesting that the positivity effect elicited by the *Status* condition, relative to the *Ambiguous* condition in 600–900 ms window continued to develop and sustained until the end of the noun following the pronoun. There was a marginally significant two-way interaction between experimental condition and EQ, *F*_(2, 58)_ = 2.87, *p* = 0.07, ɳ${}_{p}^{2}=0.09$, and a significant three-way interaction between experimental condition, EQ, and region, *F*_(4, 116)_ = 4.90, *p* < 0.01, ɳ${}_{p}^{2}=0.14$. Linear regressions in each region revealed a significant effect of EQ on the sustained effect between the *Status* and *Referent* condition in the anterior, *b* = 1.57, *t* = 4.83, *p* < 0.001, central, *b* = 1.18, *t* = 3.33, *p* = 0.001, and posterior regions, *b* = 0.94, *t* = 2.87, *p* = 0.005, suggesting that the higher the empathy of the comprehender, the larger the sustained positivity shown in the *Status* condition (Figures 3A,B).

ANOVA taking experimental condition (3 levels: *Referent, Status, Ambiguous*) and topographic variables (3 levels of Hemisphere: left, medial, right; 3 levels of Region: Anterior, Central, Posterior) as within-participant factors and Differential Score as a covariate revealed a significant interaction between experimental condition and Differential Score, *F*_(2, 58)_ = 3.29, *p* < 0.05, ɳ${}_{p}^{2}=0.08$. Regression analysis revealed a significant effect of Differential Score on the late sustained positivity between the *Status* and *Referent* condition, *b* = 0.86, *t* = 4.32, *p* < 0.001, and between the *Status* and *Ambiguous* condition, *b* = 0.89, *t* = 3.35, *p* = 0.001. These findings suggest that the differential score between the status-incongruent and status-congruent sentences in the appropriateness rating predicted the sustained positive response. Comprehenders with increased sensitivity to the appropriate usage of pronoun in different status contexts demonstrated larger positivity effects in the *Status* condition (Figures 4A,B).

To evaluate whether the empathic ability modulated the positivity effect through the status-sensitivity in the *Status* condition, mediation analyses were performed for each ROI, with empathic ability as the independent variable, the ERP magnitude difference between the *Status* and *Referent* conditions as the dependent variable, and the Differential Score in the appropriateness rating of the status-incongruent vs. congruent sentences as the mediator. We tested for mediation by deriving 95% bias-corrected confidence intervals (CIs) from 5000 bootstrap estimates (MacKinnon et al., 2004; Preacher and Hayes, 2004, 2008). Higher EQ predicted greater Differential Score, which in turn predicted greater amplitude of the late positive effects in the *Status* condition (in the left and medial posterior regions in the 600–900 ms window and in the medial posterior region in the 900–1600 ms window). The indirect path was significant (600–900 ms: *b* = 1.35, *t* = 3.19, *p* = 0.003; *b* = 1.54, *t* = 3.23, *p* = 0.003; 900–1600 ms: *b* = 1.48, *t* = 2.73, *p* = 0.01), and the estimates of the direct path between EQ and the amplitude of the positive response were reduced but still marginally significant when the mediator was entered in the model (600–900 ms: *b* = 1.15, *t* = 1.91, *p* = 0.06; *b* = 1.32, *t* = 1.96, *p* = 0.05; 900–1600 ms: *b* = 1.28, *t* = 1.76, *p* = 0.09), suggesting that the status-sensitivity partially mediated the relationship between EQ and the late positive effects. The robustness of the mediation effect testing CIs (at 95% level) confirmed the mediator role of the status-sensitivity [600–900 ms: (0.02, 1.46); (0.05, 1.90); 900–1600 ms: (0.01, 1.71)]. These findings indicate that comprehenders with a higher EQ had increased positive responses in the *Status* condition and this effect was partly due to the increased status-sensitivity of these individuals.

#### The late, anterior negativity effect: 1300–1600 ms time window

ANOVA with experimental condition (3 levels: *Referent, Status, Ambiguous*) and topographic variables (3 levels of Hemisphere: left, medial, right; 3 levels of Region: Anterior, Central, Posterior) as within-participant factors and EQ as a covariate revealed a significant three-way interaction between experimental condition, Hemisphere, and Region, *F*_(8, 232)_ = 2.98, *p* < 0.05, ɳ${}_{p}^{2}=0.09$. Further analysis for each ROI revealed marginally significant differences between the *Ambiguous* and *Referent* conditions in the left anterior, medial anterior, and right anterior regions, *ps* < 0.05, and between the *Status* and *Referent* conditions in the left central, and left posterior regions, *ps* < 0.05. As shown in Figures 1, 2, these findings suggest that the *Ambiguous* condition elicited an anteriorly-distributed negativity effect, relative to the *Referent* condition, while *Status* condition elicited a larger posterior positivity. ANOVA also revealed a marginally significant interaction between EQ and experimental condition, *F*_(2, 58)_ = 3.04, *p* = 0.07, ɳ${}_{p}^{2}=0.09$, and a significant three-way interaction between EQ, experimental condition and Region, *F*_(4, 116)_ = 5.63, *p* < 0.01, ɳ${}_{p}^{2}=0.16$. Linear regression analysis did not reveal any effect of EQ on the ERP differences between the *Ambiguous* and the *Referent* conditions, *ps* > 0.1, but it did reveal an effect of EQ on the difference between the *Status* and the *Referent* conditions in the anterior, *b* = 1.60, *t* = 4.61, *p* < 0.001, central, *b* = 1.18, *t* = 3.26, *p* = 0.002, and posterior regions, *b* = 1.02, *t* = 2.86, *p* = 0.005. Such a predictability effect of EQ, consistent with the previous analysis of the ERP effects in the 600–900 ms and 900–1600 ms windows, suggests a continuous impact of the comprehender's empathic ability on ERP responses elicited by the *Status* condition. ANOVA with experimental condition and topographic variables as within-participant factors and Differential Score as a covariate found only a marginally significant three-way interaction, *F*_(4, 116)_ = 3.26, *p* < 0.05, ɳ${}_{p}^{2}=0.07$. However, further analysis did not reveal any significant effect of Differential Score on the negativity for the *Ambiguous* condition.

## Discussion

This study aimed to provide behavioral and neurophysiological evidence on how the social status information in the context and the individual's empathy and sensitivity to social status affect referential ambiguity resolution in directly-quoted utterances. We first demonstrated a graded decrease of the perceived ambiguity over the *Ambiguous, Status*, and *Referent* conditions. The perceived ambiguity was the lowest in the *Referent* condition, suggesting that a status word before the second-person pronoun can serve as a cue for the reactivation of the target referent and effectively facilitate pronoun resolution. The ambiguity in the *Status* condition was also lower than that in the *Ambiguous* condition; the comprehender may compare the social status of the two potential referents in the context and choose the one of higher status for the respectful pronoun in the former condition. In the *Ambiguous* condition, however, the two potential referents in the context were of equal status and they engaged in a dead-end competition for being the target addressee of the pronoun, which involved an effortful maintenance of the antecedent information in the limited working memory.

Consistent with the hypothesis that empathic ability plays a crucial role for comprehenders to make use of pragmatic (social status) information in the context to resolve referential ambiguity, we demonstrated that EQ modulates the perceived ambiguity in the Status condition: individuals' with higher EQ perceived less ambiguity in the sentences even when a clear social status difference existed between the two potential referents. This hypothesis was further supported by the finding that the comprehender having higher sensitivity to social status information also perceived less ambiguity in the Status condition, suggesting that individuals sensitive to the social status hierarchy are more able to resolve referential ambiguity using the status information.

Findings in the ERP data are generally consistent with the above arguments. In the following discussions, we focus on the ERP effects for different experimental conditions.

### The increased N400 responses in the referent condition

Generally speaking, the N400 responses are reduced in a highly-predictive sentential or discourse context in which the mental representation of contextual information or an individual lexical item facilitates the semantic access of the target word (e.g., “access account,” Kutas and Federmeier, 2011); the N400 responses are enhanced when a target word is incongruent with the previous lexical, sentential, or conversational context or is difficult in being integrated into the comprehender's knowledge or belief system (“integration account,” Hagoort et al., 2004; Van Berkum et al., 2009; Leuthold et al., 2012; Nieuwland and Martin, 2012; Jiang et al., 2013a,b; Ellis et al., 2015; Wang et al., 2015). A respectful pronoun used to address a lower-status addressee or a less respectful pronoun used to address a higher-status addressee elicited increased N400 responses (Jiang et al., 2013b).

The first account (Kutas and Federmeier, 2000, 2011) argues that the sentence context with more constraining information toward the upcoming word reduce N400 responses to that word. In the *Referent* condition, the additional status word preceding the pronoun formulates additional contextual information which may reduce the N400 response and ease the access toward the upcoming respectful pronoun. The behavioral rating revealed a lower perceived ambiguity in the *Referent* condition than the other two conditions. However, we found that the pronoun in the *Referent* condition showed larger N400 responses than those in the other conditions, a pattern opposite to what would be predicted by the access account; this account would predict an easier rather than a disruptive access for the *Referent* condition. Alternatively, given that there were 90 critical scenarios with *Nin-de* as the target pronoun but only 40 filler scenarios with *Ni-de* as the target pronoun, the system might be biased toward expecting the higher-status individual as the potential addressee. Such expectancy would reduce the N400 responses to *nin-de* in all the conditions relative to a balanced design, but less so for the *Status* and *Ambiguous* conditions. This would lead to enlarged N400 effects for the two condition, compared to the *Referent* condition, a prediction, however, not supported by our data.

The integration account attributes the increased N400 responses in the *Referent* condition, relative to the other two conditions, to the increased effort of simultaneously integrating the pronoun to the higher-status referent and to the status word inserted before the pronoun. The modulation of N400 has been found on the pronoun with no explicit antecedent in the context to be integrated with (e.g., *the in-flight meal I got was more impressive than usual. In fact*, *he/they* *courteously presented the food as well*.). The pronoun (*he*) that highly demands an explicit antecedent elicited larger negative responses than the one (*they*) that is less disruptive in the absence of the antecedent (Filik et al., 2008). Another study required the listeners to discriminate a visually presented object from its competitor based on the auditory description of its color and shape. The N400 observed on the color word (e.g., “*red*” in *the red square*) was increased when this word was redundantly uttered for discrimination in the visual display (e.g., *a red square and a blue star*), relative to when it served as critical information (e.g., *a red square and a blue square*, Engelhardt et al., 2011). These findings suggest that the N400 increase is associated with the increased demand of integration between the referential expression and what it is referred to in the context. Here, although the status word could help to disambiguate which of the two characters in the context should be the addressee and make the reference tracking easier, the pronoun nevertheless has to be linked with both the status word in the utterance and one of the characters described in the context. An integrated representation of “whom is referred to” has to be established based on the context including both the character and the status word. Such integration effort was reduced in the *Status* condition since the pronoun merely linked with the character of higher status, which had been specified by the character's name.

Future studies can better address how the pronoun-locked N400 effect is affected by the conversational context by adding a control condition which includes an ambiguous context and an explicit status word, and by comparing the unambiguous *Referent* condition with that control condition. The integration account would still predict a larger N400 in the unambiguous than the ambiguous condition due to the necessity to link the pronoun with both the status word and the contextually appropriate antecedent. The access account would predict a reduced N400 for the former due to facilitated access of a higher-status antecedent in the context.

How then can we account for the correlations between the N400 effect and the individuals' empathic ability or sensitivity to social status? Previous studies have shown that nouns mismatching the pragmatic constraints of scalar quantifiers elicited an N400 effect only in readers with a low autistic quotient (Nieuwland et al., 2010); pronouns mismatching the social status in the context elicited an N400 effect only in readers exhibiting high fantasizing ability (Jiang and Zhou, 2015). Different components of cognitive empathy (i.e., perspective-taking and fantasizing) differentially modulated the neural activity underlying pragmatic failure (which demanded a re-interpretation/conflict resolution) and pragmatic under-specification (which demanded an inferential process) (Li et al., 2014). Based on these findings, one can envisage that the comprehenders' sensitivity to the social status information in the communicative context or their empathic ability in deriving the underlying message could modulate the processes in making use of the status information and specifying an appropriate antecedent for the pronoun or in dealing with pragmatic ambiguity. The stronger the ability, the weaker the N400 responses to the pronoun in the *Status* or the *Ambiguous* conditions, and the larger the N400 effect for the *Referent* condition. In other words, the variation of the size of the N400 effect over participants was mainly due to individual differences in the neural responses to the *Status* and *Ambiguous* conditions rather than the neural responses to the *Referent* condition.

Similarly, comprehenders with increased sensitivity to the status-pronoun mismatch showed a larger N400 effect when the pronoun mismatched the social context, replicating Jiang and Zhou (2015). In the latter study, the readers were presented with scenarios in which the informal form of the second-person pronoun was used to address the addressee of lower status (correct usage) or the addressee of higher status (disrespectful usage). The N400 responses were enlarged on pronouns used in a disrespectful way, and this effect was modulated by the comprehenders' Difference Score ratings for the appropriateness of the respectful and disrespectful scenarios. In the current study, the successful resolution of the pronoun in the directly-quoted utterance depended on the matching of the respectful pronoun with the person of higher social status in the context. The higher the sensitivity to the social status information, the stronger the ability to use this information, the weaker the N400 responses to the pronoun in the *Status* or the *Ambiguous* conditions, and the larger the N400 effect for the *Referent* condition.

It should be noted that status-sensitivity and empathic ability modulated the N400 effect in different hemispheric sites: the effect of status-sensitivity was in the right hemisphere, whereas the effect of the empathic ability was in the left and medial hemispheric sites. Individuals who excelled in recognizing social status in the context and those who showed expertise in empathizing the conversational partner may engage different neurocognitive mechanisms in integrating the pronoun with the context, although further studies are needed to elucidate these mechanisms.

### The late, sustained positivity in the status condition

The *Status* condition elicited a positivity effect post-onset on the pronoun that sustained until the end of the following object noun. This effect was similar to the positivity (P600) found on words eliciting ironic interpretations (Regel et al., 2010, 2011; Spotorno et al., 2013; Filik et al., 2014). This effect was also similar to the sustained positivity effect found on the respectful pronoun (and the word immediately following the pronoun) when the pronoun was used sarcastically in a directly-quoted utterance (i.e., used by a higher-status speaker addressing a lower-status addressee, Jiang et al., 2013b). A sustained positivity effect has also been found on words inconsistent with the preceding context describing an individual's traits, intention, or goal of an action (Van Duynslaeger et al., 2007; Baetens et al., 2011); it has been suggested to be manifested by the neural network subserving the mentalizing process (e.g., the temporo-parietal junction, Van Duynslaeger et al., 2007). These positivity effects may reflect a “pragmatic enrichment,” second-pass processing strategy when a literal interpretation of the input meets difficulties and when contextual cues are sufficient to allow for the use of this strategy (Xu et al., 2015). Positivities with different latencies may subserve different components of pragmatic inference. The P600-like effect (340–730 ms) was found in vocal expressions which were ambiguous in indicating a speaker's confidence, while a more delayed sustained positivity was found in neutral-intending expressions which were acoustically different but perceptually similar to the confident expressions (Jiang and Pell, 2015); the former was associated with the attempt of continued evaluation of an ambiguous input, and the latter was responsible for successful derivation of the speaker's meaning from an incongruent perception. In the *Status* condition of the current study, the late positivity (in 600–900 ms) and the delayed sustained positivity (900–1600 ms) may reflect different sub-processes. The comprehender was faced with a temporary referential ambiguity which may require continued analysis (the late positivity); this ambiguity was eventually resolved by the pragmatic inference process that was based on the status information in the context and the usage of respectful pronoun (the sustained positivity). This account is consistent with the MRC (Mental Representation of what is being Communicated) model suggested by Brouwer et al. (2012) and Brouwer and Hoeks (2013). Here the positivity effect could be interpreted as reflecting the difficulty of integrating the pronoun into the mental representation pre-established according to the communicative context. The context specifies two potential addressees, and an inference process must be conducted to establish which one is the actual addressee that could be linked with the pronoun. Only through this process can the pronoun be integrated into the MRC.

The positivity effects were modulated by the comprehender's empathic ability: the comprehender with higher empathizing ability displayed a larger positivity effect. This finding suggested that those who excel in empathizing are more likely to initiate the effort of inferring the addressee using the social status information in the context or the effort of integrating the pronoun into the communicative context. Such effort may help to reduce the ambiguity created by two potential antecedents. Indeed, we showed in the behavioral data that the increased EQ scores were associated with decreased perceived ambiguity when the context biases the selection of the target addressee. Another possibility is that the high-empathy group have higher sensitivity to the pragmatic cues such as the social status of the communicator (Van den Brink et al., 2012) and this sensitivity facilitates the selection of the target addressee based on the context biases. These findings provide a first piece of evidence showing that using contextual information to implement pragmatic inference is subject to individual's empathic ability in resolving verbal ambiguity (Li et al., 2014). In line with this argument, the EQ modulated the appropriateness rating of the respectful form usage, demonstrating its impact on individual's sensitivity to the social status of the addressee. Moreover, the mediation analysis confirmed that the empathic ability affected the positivity effect partially through individuals' sensitivity to the status information in the context.

### The delayed anterior negativity in the ambiguous condition

An early-starting, anteriorly distributed sustained negativity effect (*Nref*) has been observed on the third-person pronoun when two competing characters in the discourse are equally likely to be the antecedent of this pronoun (Van Berkum et al., 1999; Nieuwland and Van Berkum, 2006; Nieuwland et al., 2007). Different from the previous studies, the ambiguity in this experiment was developed on the second-person pronoun in a directly-quoted utterance whose referent had to be determined based on the social status information. The starting portion of *Nref* for the *Ambiguous* condition (relative to a one-referent, unambiguous baseline) may have overlapped with the N400 effect for the *Referent* condition, preventing us from observing this effect. However, assuming that the integration cost for the additional status word in the *Referent* condition had dissipated in the time windows later than the N400 window, the *Nref* for the *Ambiguous* condition, relative to the *Referent* condition, would be observable. The competition between the two possible referents would last for a long time until new information comes to specify which is a more possible antecedent of the pronoun.

## Conclusion

This study examined the role of social context as well as individual differences in ambiguity resolution during the comprehension of conversational scenario involving a directly-quoted utterance and a singular, respectful pronoun. Behaviorally, the perceived ambiguity gradually decreased over the scenario without a disambiguating cue (the *Ambiguous* condition), the scenario in which differential status between the potential referents bias one to be the target addressee (the *Status* condition), and the scenario in which a status word unambiguously vocalized the target addressee (the *Referent* condition). Comprehenders with increased status-sensitivity perceived less ambiguity in the *Status* condition and more ambiguity in the *Ambiguous* condition; comprehenders with higher empathic ability also perceived less ambiguity in the *Status* condition. Electrophysiologically, the *Referent, Status*, and *Ambiguous* conditions were distinctively captured by increased N400 responses (300–600 ms), increased late sustained positivity (600–1600 ms), or late anterior negativity (or *Nref*, 1300–1600 ms), demonstrating differential neurocognitive processes underlying ambiguity resolution with different contextual cues. The late positivity effect demonstrated an inferential process in which pragmatic information was used to establish a potential referential link between the pronoun and its antecedent. The late negativity effect demonstrated an increased computational load in choosing one of the two competing antecedents. These findings highlight the role of disambiguating cues in the social context and the neurocognitive flexibility in using these cues to establish referential representations during utterance comprehension.

### Conflict of interest statement

The authors declare that the research was conducted in the absence of any commercial or financial relationships that could be construed as a potential conflict of interest.
